# Clinical Biomarker of Sterile Inflammation, HMGB1, in Patients with Chronic Non-Specific Low Back Pain: A Pilot Cross-Sectional Study

**DOI:** 10.3390/life13020468

**Published:** 2023-02-08

**Authors:** Julita A. Teodorczyk-Injeyan, Heba Khella, H. Stephen Injeyan

**Affiliations:** 1 Graduate Education and Research Programs, Canadian Memorial Chiropractic College, Toronto, ON M2H 3J1, Canada; 2 Department of Clinical Education, Canadian Memorial Chiropractic College, Toronto, ON M2H 3J1, Canada

**Keywords:** HMGB1, sterile inflammation, low back pain

## Abstract

The present study explores whether the inflammatory biomarker of sterile inflammation, high mobility box 1 (HMGB1), contributes to the inflammatory/nociceptive pathophysiology that characterizes chronic non-specific low back pain (LBP). Patients with chronic LBP (N = 10, >3 pain score on a 11-point Visual Analogue Scale, VAS) and asymptomatic participants (N = 12) provided peripheral blood (PB) samples. The proportion of classical CD14++ monocytes within PB leukocytes was determined by flow cytometry. The plasma and extracellular HMGB1 levels in unstimulated adherent cell (AC) cultures were measured using specific immunoassays. HMGB1 localization in ACs was assessed by immunofluorescent staining. The relative gene expression levels of tumor necrosis factor α (TNFα), interleukin-1 beta (IL-1β) and HMGB1 were determined by quantitative polymerase chain reaction (qRT-PCR) in relation to the pain intensity (11-point VAS scores) in patients with LBP. The extracellular release of HMGB1 in the LBP patient AC cultures was significantly elevated (*p* = 0.001) and accompanied by its relocation into the cytoplasm from the nuclei. The number of CD14++ monocytes in the patients’ PB was significantly (*p* = 0.03) reduced, while the HMGB1 plasma levels remained comparable to those of the controls. The mRNA levels of TNFα, IL-1β and HMGB1 were overexpressed relative to the controls and those of HMGB1 and IL-1β were correlated with the VAS scores at a significant level (*p* = 0.01–0.03). The results suggest that HMGB1 may play an important role in the pathophysiology of chronic non-specific LBP.

## 1. Introduction

Low back pain is one of the most commonly experienced conditions by people worldwide [1,2]. While some aspects of low back pain are clearly clinically and radiologically identifiable, and in rare cases may lead to surgery, most remain vague, with no specific underlying pathophysiological mechanism, and are recognized to result from a combination of interacting biophysical and psychosocial factors. Hence, they are referred to as being non-specific [2]. Recent research studies indicate that non-specific low back pain (LPB), as well as other forms of spinal pain, are associated with the activation of the cellular components of the innate (inflammatory) immune response. The systemic presence of classical pro-inflammatory cytokines, such as tumor necrosis factor α (TNFα), interleukin 1β (IL-1β) and other inflammatory markers, in patients with non-specific LBP support this notion [3,4]. In patients with acute and chronic non-specific LBP, a significant augmentation of the inducible (in vitro) production of monocytic pro-inflammatory mediators has also been reported [5]. The continuous activation of pro-inflammatory pathways in patients with LBP has been evidenced by observations of protractedly elevated ex-vivo production of CC series of pro-inflammatory, nociceptive cytokines (CCL2, CCL3 and CCL4), alongside increased proinflammatory to anti-inflammatory cytokine ratios [6,7].

These observations may suggest that, in patients with LBP, circulating immune cells sustain a proinflammatory state of activation. Nevertheless, the etiology of this condition remains unclear. Inflammation engaging the host’s innate immune system is commonly associated with pathogenic infections or the action of microbial products and the consequential upregulation of inflammatory genes expression in human peripheral blood mononuclear cells [8]. Although nonpyrogenic bacteria have been demonstrated in surgically removed herniated discs from low back pain patients [9], it is highly contentious that bacterial infections could contribute to the development and chronicity of non-specific LBP [10]. On the other hand, the inflammatory response of immunocytes may be elicited not only by pathogen-associated molecular pattern (PAMP), but also by damage-associated molecular pattern (DAMP), molecules derived from injured or stressed tissues [11].

Within the immune system, a prototypical DAMP molecule is the high mobility box 1 (HMGB1), a highly conserved nuclear protein that, upon its active or passive release into extracellular space, targets cells of innate and specific immunity, prompting the development of sterile inflammation [12]. Under stressful conditions, such as tissue injury, HMGB1 is synthesized and released by a variety of cells, including human monocytes [13]. The redox-state-dependent interaction of extracellular HMGB1 with different cell surface receptor(s) transduces intracellular signaling to entice the synthesis of pro-inflammatory cytokines, TNFα, IL-1β, IL-6, IL-8 [14] and also to exert chemotactic activity [15]. A myriad of physiologic activities of extracellular HMGB1 apparent in the mediation/induction of inflammatory reactions, the regulation of inflammatory cell migration, tissue damage/regeneration and nociception have resulted in the designation of this molecule as a non-classical inflammatory cytokine [13,16,17].

HMGB1 involvement in disc pathology has been demonstrated in specimens of disc tissue from patients requiring spinal surgery [18,19,20]; however, its possible role in the inflammatory response related to non-specific LBP has not been investigated. As a first step in this line of investigation, the present study was undertaken to examine the plasma levels of HMGB1, as well as its intracellular localization and extracellular secretion in cultures of peripheral blood monocytes from patients with chronic nonspecific LBP. Furthermore, the gene expression levels of HMGB1, TNFα and IL-1β, in peripheral blood leukocytes and their relationship to LBP patients’ VAS scores were examined.

## 2. Methods

### 2.1. Participating Subjects

The present investigation is an extension of a previously conducted study, which has been published [5,6]. It was approved by the Research Ethics Board of the Canadian Memorial Chiropractic College. Ten patients with chronic LBP (3 months in duration or longer) aged 22–58 years were enrolled through the CMCC outpatient clinics between January 2018 and April 2019 (Table 1). The participant recruitment and eligibility criteria were as follows: the inclusion criteria were a diagnosis of non-specific LBP based on history and physical examination performed by, or under the supervision of, experienced clinicians of the outpatient clinics. Chronicity was defined as pain being 12 weeks in duration or longer (L1–L5 spinal segments, with or without sacroiliac involvement), age 22–60 years, pain level of >3 on a 11 point-visual analog scale (VAS). Patients were excluded if they fell outside the age limits of 22 to 60 years, had a pain level below VAS 3, exhibited radicular symptoms, had taken anti-inflammatory medications in the preceding 48 h, had any type of unresolved known inflammatory condition including musculoskeletal complaints other than the presenting LBP condition, autoimmune diseases, metabolic disorders, coagulopathies, infections or neoplastic diseases, psychological disorders, were pregnant, or unwilling to sign the study consent form. Radiographic examination of the participants was not clinically warranted and was not carried out solely for the purpose of the study. A cohort of age-matched and sex-matched healthy asymptomatic participants were recruited from the general population to serve as the control group. The same exclusion criteria, and the absence of any LBP for a minimum of one year, applied in this recruitment process. All participants signed the study informed consent form.

### 2.2. Laboratory Studies

#### 2.2.1. Blood Collection and Processing

At initial presentation and prior to any therapeutic intervention, heparinized blood samples (20 mL each) were obtained by venipuncture from the cubital fossa area of a participant’s arm. The samples were transferred to the research laboratory and processed immediately. An aliquot of 3 mL of blood was used for the determination of the plasma levels of HMGB1, while 2 mL of blood was retained for the phenotypic studies. The plasma samples were prepared by centrifugation (15 min, 3000× *g*, 4 °C), aliquoted and stored at −80 °C until further studies. Peripheral blood mononuclear cells (PBMC) were prepared from the remaining 15 mL of blood by conventional density gradient centrifugation over endotoxin pre-tested (<0.12 EU/mL) Ficoll-Paque^TM^ PLUS (GE Healthcare Bio-Science AB, Uppsala, Sweden). The cells collected from the interface (PBMCs), thoroughly washed and divided to be used for: (a) preparation of adherent cell (AC) cultures; (b) cryopreservation at −80 °C in RNA*later*^TM^ solution (Invitrogen, by Thermo Fisher Scientific Baltics, UAB, Vilnius, Lithuania). To prepare the AC cultures, the PBMCs were resuspended at the concentration of 2.5 × 10^6^/mL in a complete tissue culture medium (TCM) consisting of RPMI 1640 supplemented with 5%, heat inactivated, fetal bovine serum preselected in regard of low endotoxin level (HyClone/Cytiva, Global Life Sciences Solutions ULC, Canada), 5 × 10^−5^ M 2-mercaptoethanol (Sigma, St. Lois, MO, USA) and antibiotics (Pen-Strep, Gibco, Life Technologies, Grand Island, NY, USA).

#### 2.2.2. Immunophenotype Staining

To determine the total number of monocytes bearing a classical CD14^++^ phenotype, a triple color analysis of the monocyte surface receptors was performed. The samples of PB from the studied patients and controls were first incubated with Fc receptor (FcR) blocking solution (BioLegend, San Diego, CA, USA). The FcR-blocked sample aliquots were then stained with the following fluorochrome-labeled monoclonal antibodies: PE-conjugated anti-CD14, FITC-conjugated anti-CD195, and PerCP/Cy5.5a-conjugated anti-CD192 (all from BioLegend, San Diego, CA, USA). The parallel PB samples were left unstained to create fluorescence minus one (FMO) controls. These, in addition to compensation/viability controls, were also prepared according to the protocol established by the Flow Cytometry Facility at the University of Toronto (Department of Immunology, Toronto, ON, Canada). All of the samples were fixed in paraformaldehyde and processed by Flow Cytometry (BD Biosciences, LSR Fortessa^TM^ cell analyser) within 24 h of blood collection. Analyses of the flow cytometry data was performed using the Flow Jo software (FlowJo LLC, Ashland, OR, USA). Briefly, the peripheral blood mononuclear cells were displayed according to side scatter (SSC-A) and forward scatter (FSC-A), and the monocytes were selected. Singlet exclusion was carried out using FCS-H vs. SS-H vs. SSC-W. Live, classical CD14++ (PE) were gated according to the expression of CD192 (PerCP/Cy5.5a) and CD195 (FITC). The FMO controls were used for all of the surface markers gating to properly identify antigen-positive cells.

#### 2.2.3. HMGB1 Determination in Plasma and TC Supernatants

Determinations of the HMGB1 concentrations in the plasma, as well as in the TC supernatants of the studied preparations, were performed using human HMGB1 enzyme-linked immunosorbent assay (ELISA) kits, strictly according to manufacturer’s instructions (My BioSource, San Diego, CA, USA). The plasma samples were examined initially without processing. Briefly, the standards and samples were incubated in microplates pre-coated with a biotin-conjugated specific anti-HMGB1 antibody. Following the removal of all liquid, the wells were exposed to an avidin-conjugated HRP (horseradish peroxidase) solution. They were then washed with washing buffer, and TMB substrate solution was added. The reaction was terminated by the addition of sulfuric acid solution. The absorbance of the developed color was measured at λ = 450 nm with λ = 570 nm correction filter using a multichannel spectrophotometer (Epoch Bio-Tech, Winooski, VT, USA). The concentrations of HMBG1 in the tested samples were determined using Gen5 Data Analysis Software (Bio-Tech) and the detection limit for the assay was 62.5 pg/mL.

To increase the sensitivity of the detection of HMGB1 in the plasma, the samples were treated with perchloric acid (PCA) at the final concentration of 3% and processed strictly as described in detail by Gaillard et al. [21]. Assays were performed at a sample dilution of 1/100 in phosphate buffered saline (PBS) according to the manufacturer’s instructions. Duplicate aliquots of the AC culture supernatants, as well as untreated and PCA-treated plasma samples, were tested at least twice.

#### 2.2.4. Preparation of Adherent Cell Cultures and Immunofluorescent Staining

The AC cultures were prepared using single-well chamber slides, specially treated for adherent cells (Thermo Fisher Scientific Nunc™ Lab-Tek™ II Chamber Slides). The PMBC suspensions from the patients with LBP and from the asymptomatic controls were placed in chambers at a volume of 2.5 mL/chamber and incubated for 2 h at 37 °C. Following incubation, non-adherent cells were removed by triple washing in warm TCM. The chambers were then filled with 2 mL of fresh TCM and cultured overnight at 37 °C in a 5% CO_2_ incubator. At the termination of the incubation period, the culture supernatants were collected, centrifuged to remove cellular debris, aliquoted and placed at −80 °C for subsequent analyses. The cultured AC were then washed gently with Ca^2+^, Mg^2+^ PBS and processed for immunofluorescent staining based on the method described by Bonaldi et al. [22]. Briefly, the cells were fixed in 4% phosphate paraformaldehyde (PFA) and permeabilized with ice-cold methanol. Following washing and incubation in blocking reagent (BR; 0.2% BSA), a primary mouse monoclonal anti-human HMGB/HMG-1 antibody (R and D Systems, Minneapolis, MO, USA), diluted in BR to a working concentration of 10 μg/mL, was applied for 2 h at room temperature. Goat-anti-mouse polyclonal AlexaFluor^R^488 (Abcam, Cambridge, MA, USA), at a dilution of 1:500 in BR, was used as the secondary antibody. To visualize the nuclei, the slides were mounted with Molecular Probes^R^ SlowFade Diamond Antifade Mountand with 4′,6-diamidino-2-phenylindole (DAPI) (Thermofisher Scientific). Images were attained using Zeiss Axioscope.A1 and AxiCam ICm1 camera (Jena, Germany) and assessed independently by 2 investigators to determine, semi-quantitatively, the proportion of cells displaying altered nuclear localization and extracellular manifestation of HMGB1.

#### 2.2.5. Quantitative RT-PCR (qRT-PCR)

Total RNA isolation from the frozen PBMC samples was carried out using a Trizol Plus Purification Kit (Thermo Fisher Scientific, Mississauga, ON, Canada), according to the manufacturer’s protocol. The cells were harvested by centrifugation and lysed and homogenized by the addition of Trizol™ Reagent. After 5 min incubation, chloroform was added, followed by a brief incubation for 2–3 min. The samples were centrifuged and the aqueous upper phase containing RNA was transferred to a new tube. Ethanol was added, and the samples were transferred to a spin cartridge, followed by washing in the appropriate buffers, provided by the manufacturer. The RNA quality and concentrations were determined spectrophotometrically using NanoDrop 2000^TM^ (Thermo Fisher Scientific, Mississauga, ON, Canada). The samples to be used for analyses were stored at −80 °C. Reverse transcription reaction was performed using the high-capacity RNA-to-cDNA kit (Thermo Fisher Scientific) according to the manufacturer’s instructions. A reverse transcriptase (RT) reaction mix was prepared and aliquoted into 48 well reaction plates. The RNA samples were added, the plates were sealed, briefly centrifuged, and placed in a thermal cycler for processing, according to the manufacturer’s instructions. Gene expression analysis of the HMGB1, TNFα and IL-1β was performed by qRT-PCR, using predesigned TaqMan Gene assays on the Step One™ Real-Time PCR System (Thermo Fisher Scientific). Glyceraldehyde 3-phosphate dehydrogenase (GAPDH) was utilized as the endogenous control. A PCR reaction mix was prepared and aliquoted into reaction plates, cDNA was added, and the plates were sealed and centrifuged. The thermal cycling conditions were as specified by the manufacturer’s protocol and all reactions were performed in duplicate. The fold change was determined using the 2-^ΔΔC^ T method [23].

## 3. Statistics

The statistical significance of the data was assessed using the PAST 3.18 beta software [24] and GraphPad Prism 9 (GraphPad Software, San Diego, CA, USA). The data attained for both study groups were tested for normality using the Shapiro Wilk test and if non-normal distributions were found, the data were transformed (Box-Cox) and the analyses were repeated. The primary outcome measures for this study were determined as the differences in the levels of extracellular HMGB1 release, the quantitative changes in the level of peripheral blood monocytes, and the differences in the mRNA expression levels between the asymptomatic (control) participants and the patients with LBP. Differences between the first two of the aforementioned outcomes in the patients with LBP vs. asymptomatic subjects were also assessed using a standard deviation metric, calculated as Cohen’s *d/SD_pooled_*. Pearson’s correlation coefficients and their statistical significance were determined to assess the relationship between the pain scores (VAS) and mRNA expression levels. Testing for differences between the control and patients with LBP was performed using an independent samples *t* test. A non-parametric Mann-Whitney U test was also used for the data without normal distribution and to confirm the results. Unless indicated, the results are presented as means ± SD. Values *p* ≤ 0.05 were accepted as being significant.

## 4. Results

The demographic data (Table 1) showed that the patients with LBP were somewhat older than those in the control group (35.2 ± 13 vs. 27.6 ± 3). However, this difference was not statistically significant. Similarly, the mean values of the body mass index (BMI) did not differ between the two groups.

BMI: body mass index; VAS: 11-point visual analogue scale.

### 4.1. Determinations of Extracellular HMGB1 Levels

#### 4.1.1. Plasma

The HMGB1 levels, both in the unprocessed and the PCA-treated plasma samples from the LBP patients, were not significantly different from the control levels (0–6.2 ng/mL).

#### 4.1.2. AC Culture Supernatants

**Figure 1:**
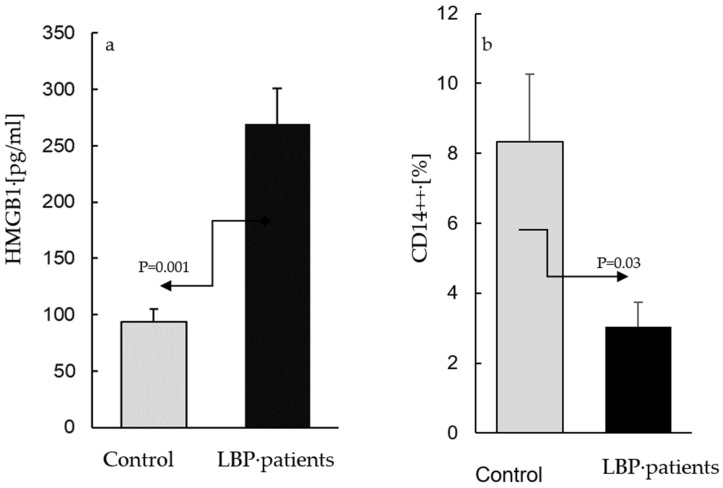
The levels of extracellular HMGB1 in supernatants of adherent cell (AC) cultures from asymptomatic subjects (control) and patients with low back pain (**a**) and the mean percentages of total CD14++ monocytes in blood samples used for preparation of control and patients’ cultures (**b**). (**a**). ACs were prepared as described in the Material and Methods section and were cultivated for 24 h without stimulation. The mean level of extracellular HMGB1 (±SEM) in supernatants of AC cultures from patients with LBP is significantly higher (*p* = 0.001) than that in the control cultures. (**b**) Compared with the control, the number of peripheral CD14++ monocytes is significantly lower (*p* = 0.03) in patients with LBP.

The AC culture supernatants from the asymptomatic participants contained HMGB1 at concentrations between 65–173 pg/mL, while those from the cultures derived from the patients with LBP ranged between 80–482 pg/mL. Figure 1 depicts the means of the HMGB1 levels in the LBP patients and the controls: (a) alongside the means of the percentages of CD14++ cells determined within the population of the peripheral blood leukocytes of the same subjects; (b) compared with the control, the levels of HMGB1 were significantly elevated (*p* = 0.001) in the culture supernatants from the patients with LBP (269 ± 105 vs. 94 ± 48 pg/mL, respectively, Figure 1a). In contrast, the fraction of classical CD14++ monocytes in the blood samples used for the preparation of the AC cultures from the LBP patients was significantly (*p* = 0.03) lower than that in the peripheral blood of the asymptomatic controls (3.4% ± 2.3 vs. 8.5 ± 4.1%, respectively, Figure 1b). The differences between the levels of extracellular HMGB1 release, as well as the number of the peripheral blood CD14++ monocytes in the LBP patients vs. asymptomatic subjects. were large (Cohen’s *d/SD*: 2.1 and 1.9, respectively).

### 4.2. Immunofluorescent Staining of Adherent Cells (AC)

**Figure 2:**
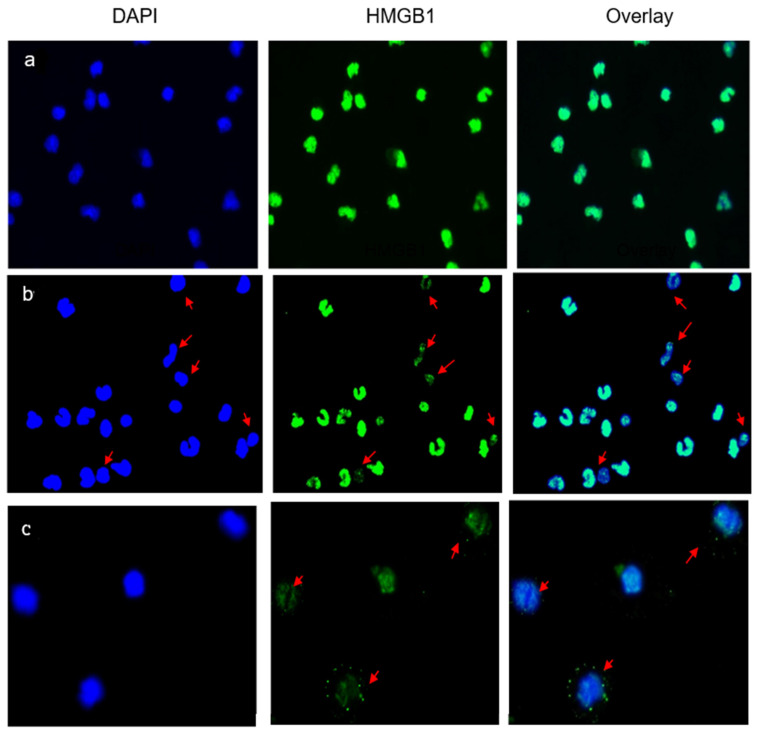
Nuclear and extracellular localization of HMGB1 in the studied subjects. (**a**) A typical representation of immunofluorescent staining of HMGB1 in AC cultures from an asymptomatic subject. HMGB1 is localized within nuclei and no cytoplasmic relocation or extracellular release are observed following the 24 h incubation period. (**b**,**c**) Immunofluorescent staining of HMGB1 in AC cultures from two representative patients with LBP. Arrows indicate ACs from which nuclear HMGB1 was actively released into the culture supernatant as depicted by DAPI+ cells devoid of/or displaying diminished contents of nuclear HMGB1 (**b**), or HMBG1 being relocated/shifted into the cytoplasm (**c**). Zeiss, N-Achroplan: (**a**,**b**) 10 ×/0.25; (c) 20 ×/0.45.

Immunofluorescence studies were used to assess the location of intracellular HMGB1 protein in the AC cultures. In control preparations (Figure 2a), the nuclei (DAPI, blue) were stained strongly with an anti-HMGB1 antibody (green), without visible staining of the cytoplasm. HMGB1 was expressed and contained within the nuclei. The nuclear expression of HMGB1 was evidently diminished in 8/10 patients with LBP and was observed in 30–50% of the enumerated ACs (Figure 2b). Typically, the protein relocated into the cytoplasm or was not observed in the nuclei (see arrows in Figure 2b,c). This morphology signposted the extracellular release of HMGB1 from the patients’ ACs into the culture medium.

### 4.3. RT-qPCR

**Figure 3:**
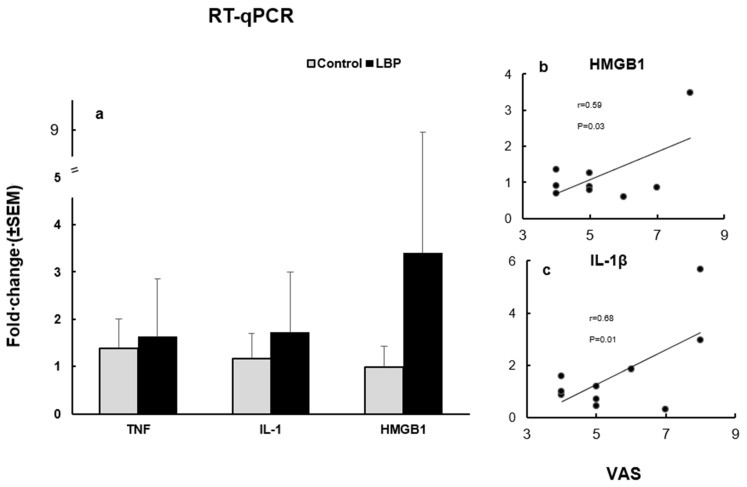
The expression levels of TNFα, IL-1β and HMGB1, at the mRNA level (**a**) and their relation to VAS scores in LBP patients (**b**). (**a**) TNFα, IL-1β and HMGB1 mRNA expression levels in the studied subjects. All three cytokines were overexpressed in patients with LBP compared to the control groups. (**b**) The correlation between VAS and the HMGB1 fold change in LBP patients was statistically significant (Pearson’s *r* = 0.59, *p* = 0.033). Outlier, with a change fold of >20, is not depicted. (**c**) The correlation between VAS and the IL-1β fold change was also statistically significant (Pearson’s *r* = 0.68, *p* = 0.014).

The mean expression levels of TNFα, IL-1β and HMGB1, at the mRNA level, in the LBP patients were compared to the control group (Figure 3a). The TNFα expression level was increased in the LBP patients compared to the control group, with fold changes up to 3.2 versus 2.0, respectively. The overexpression of IL-1β was also noticed in the LBP patients compared to the control group, with an up to 5.7 versus 2.5-fold change, respectively. The HMGB1 expression level was markedly elevated in the LBP patients compared to the control group, presenting an up to 20.7-fold change in the LBP patients versus a 1.6-fold change in the controls. However, due to the considerable variability of the values within the patient group, the observed differences did not reach statistical significance. On the other hand, the correlation between pain (VAS scores) and the HMGB1 fold change in the LBP patients was statistically significant (Pearson’s *r* = 0.5996, 95% CI: 0.04828 to 0.8923; R^2^ = 0.3595; *p* = 0.0335) (Figure 3b). Similarly, there was a significant positive correlation between pain level and the IL-1β fold change in the patients with LBP

(Pearson’s *r* = 0.6876, 95% CI: 0.1022 to 0.9193; R^2^ = 0.4487; *p* = 0.017) (Figure 3c).

## 5. Discussion

This pilot investigation demonstrates that HMGB1, a DAMP molecule and a biomarker of sterile inflammation, may be involved in the pathophysiology of LBP. In comparison with asymptomatic controls, well-matched in terms of age and BMI (Table 1), highly elevated levels of extracellular HMGB1 were detected in the culture supernatants of unstimulated adherent cells from patients with LBP (Figure 1a). The cells in the same cultures clearly displayed a relocation of nuclear HMGB1 into the cytoplasm and its release into the culture milieu (Figure 2b,c). Numerous in vitro studies have investigated the nuclear, cytoplasmic and extracellular release of HMGB1 in LPS-stimulated monocyte cultures. Importantly, the relocation of nuclear HMGB1 in the cytoplasm has been determined to be the primary step for the release of this cytokine by activated cells [25,26,27]. Monocytes are equipped with a nuclear re-shuttling mechanism and their acetylating activity controls the cellular localization of HMGB1 following inflammatory signals [22]. The present study was carried out in AC cultures incubated for 16–18 h without LPS stimulation. Despite the absence of ex vivo activation, the observed morphological changes, representative of re-location of nuclear HMGB1 and its release into the culture medium (Figure 1 and Figure 2b,c, respectively), suggest that the peripheral blood monocytes from the LBP patients have been already pre-activated in situ. Similarly, the spontaneous ex vivo release of HMGB1 has been observed in intact cultures of human osteoarthritic knee cartilage, a typical clinical model of sterile inflammation [28].

We were unable to observe significant increases in the HMGB1 levels in the patient plasma samples. However, it has been demonstrated that the assessment of HMGB1 concentrations in human plasma/serum, by even highly sensitive ELISA, might be obstructed by interfering factors, including by anti-HMGB1 IgM autoantibodies, normally present in human plasma [29]. In future studies, the utilization of more sensitive methods (e.g., Western blotting) to examine HMGB1 levels in plasma might overcome this problem.

The elevated levels of extracellular HMGB1 secreted by monocytic cells in cultures from the patients with non-specific LBP lead us to hypothesize that the enhancement of the in vitro inflammatory responses observed previously, in several studies [4,7], may be mediated by HMGB1. The data from our laboratory have shown that the LPS-induced production of the key inflammatory cytokines, TNFα, IL-1 β, IL-6, as well as nociceptive chemokines, CCL2, CCL3 and CCL-4, in whole blood cultures, and that of plasma sE-selectin in plasma, are significantly increased in patients with LBP [5,6]. HMGB1 activates human monocytes to produce pro-inflammatory cytokines and chemokines [14], and synergizes with other immunostimulatory molecules to promote inflammation [30]. It also up-regulates the endothelial cell expression and release of E-selectin [31]. The stimulatory activity of HMGB1 is comparable and synergistic with that of LPS due to their interaction with the same cellular receptor complex, TLR4 [32], and its capacity to enhance LPS-induced proinflammatory cytokine production [33]. Furthermore, HMGB1 forms complexes with proinflammatory ligands, including cytokines [34,35,36], and prompts the production of inflammatory mediators even further. Importantly, the cultures utilized in the current study were free of LPS, highlighting the contribution of HMGB1 in enhancing the production of proinflammatory mediators. These observations support the notion that the augmentation of innate immunity responses in patients with non-specific LBP may be mediated by HMGB1.

In the current study, the overexpressed HMGB1, TNFα and IL-1β levels of mRNA transcripts in the preparations of PBMCs from LBP patients suggest that at least a fraction(s) of these cells was already in a state of activation. The plasma HMGB1 levels in these patients were not significantly different from those in the asymptomatic participants. However, interpreted in light of the significantly reduced numbers of CD14++ monocytes, a major producer of this cytokine [13,14], within the PBMCs derived from the same LBP patients (Figure 1b), it may be suggested that plasma HMGB1 levels, as well as HMGB1 mRNA transcripts, might indeed be proportionately elevated. Different phases of inflammation, including sterile inflammation, have been shown to be associated with the depletion of monocytes/macrophages [37]. The preferential migration of classical monocytes expressing CD14++CD16−, CD14++CD192+ to inflamed tissues [38] and the infiltration of inflammatory cells in surgical specimens from patients with herniated lumbar discs have been also reported [39]. These observations may explain the difficulty in detecting clinically relevant changes in the systemic concentrations of inflammatory mediators in the circulation of patients with LBP, reported in this study, as well as in other studies [40].

HMGB1 is a potent mediator of both neuropathic and chronic pain [41,42,43,44] due to its capacity for enhancing pain perception via multiple toll like-receptors [45]. The present study has demonstrated that a significant correlation exists between the intensity of self-assessed pain and HMGB1 gene upregulation (Figure 3b), pointing to its nociceptive effect in non-specific LBP. It is of interest that targeting the release of HMGB1 has been considered to be a promising therapeutic modality for the treatment of a variety of inflammatory disorders, including sterile inflammation and pain [46,47]. Thus, further elucidation of the role of HMGB1 in the mediation of sterile inflammation in nonspecific LBP would be important.

## 6. Limitations

This study is preliminary and, as such, has several limitations. (1) The sample size was small due to the difficulty of recruiting patients presenting exclusively with chronic non-specific LBP. The exclusion criteria, as detailed in the methods section, were indeed highly restrictive. Furthermore, patient recruitment could not be continued beyond late-2019 because of the beginning of the COVID-19 pandemic. (2) Patients were recruited using the benchmark of symptom persistence for three months or longer. However, cross contamination with subacute cases might have occurred. (3) Although the absence of yellow and red flags was ascertained through detailed history taking at the outset, and the need of radiological assessment was ruled out through physical examination, underlying spinal degenerative changes of various degrees might have existed in some patients and may have contributed to data variability. However, such degenerative changes are common and are also expected in the general population. As such, the radiological guidelines recommend against routine imaging studies for LBP, unless clinically indicated [48] (4) Whole PBMC preparations containing reduced numbers of CD14++ cells, rather than purified (isolated) population of monocytes, were used for the gene expression studies due to the limited availability of biological material. These limitations notwithstanding, the data presented herein suggest that sterile inflammation, associated with the biologic activity of HMGB1, may induce and/or enhance the inflammatory and nociceptive responses observed in patients with non-specific low back pain. Studies addressing these limitations would be required to further explore the role of HMGB1 in the pathophysiology of nonspecific low back pain.

## 7. Conclusions

In unstimulated cultures of adherent peripheral mononuclear cells from patients with non-specific LBP, the extracellular release of HMGB1 was significantly elevated. The mRNA levels of HMGB1, TNFα and IL-1β were overexpressed relative to the controls, and those of HMGB1 and IL-1β were correlated with pain intensity. These findings indicate that HMGB1, a key DAMP molecule and prototypical cytokine, may prompt/activate proinflammatory and nociceptive pathways in patients with non-specific LBP. The role of HMGB1 in the pathophysiology of this common LBP complaint deserves to be further explored in a study(s) addressing the limitations noted above.

## Figures and Tables

**Table 1:** Demographic data and measure of pain of LBP patients participating in the study.

Mean ± SD		
Characteristics	LBP N = 10	Asymptomatic controls N = 12
Age (range)	35.2 ± 13 (22–58)	27.6 ± 3.2 (20–34)
Sex: male/female	8/2	6/6
BMI (range)	27.1 ± 3.4 (23–26)	25.5 ± 4 (20–34)
VAS (range)	5.6 ± 1.6 (4–8)	0

## Data Availability

The datasets used and/or analyzed during the current study are available from the corresponding author on reasonable request.
